# Role of Electromechanical Dyssynchrony Assessment During Acute Circulatory Failure and Its Relation to Ventriculo-Arterial Coupling

**DOI:** 10.3389/fcvm.2022.907891

**Published:** 2022-06-21

**Authors:** Stefan Andrei, Bogdan A. Popescu, Vincenza Caruso, Maxime Nguyen, Belaid Bouhemad, Pierre-Grégoire Guinot

**Affiliations:** ^1^Anaesthesiology and Critical Care Department, Dijon Bourgogne University Hospital, Dijon, France; ^2^University of Medicine and Pharmacy “Carol Davila”, Bucharest, Romania; ^3^Department of Cardiology, Euroecolab, Emergency Institute for Cardiovascular Diseases “Prof. Dr. C. C. Iliescu”, Bucharest, Romania; ^4^Department of Translational Medicine, Università del Piemonte Orientale, Novara, Italy; ^5^University of Burgundy Franche Comté, LNC UMR1231, Dijon, France

**Keywords:** electromechanical coupling, ventriculo-arterial coupling, shock, fluid therapy, inotrope, vasopressor, total isovolumic time, cardiac resynchronization

## Abstract

**Introduction:**

Two parallel paradigms of cardiovascular efficiency and haemodynamic optimisation coexist in haemodynamic research. Targeting ventriculo-arterial (VA) coupling [i.e., the ratio between arterial and ventricular elastance (E_V_)] and electromechanical coupling are two promising approaches in acute circulatory failure. However, validation of the parameters of electromechanical coupling in critically ill patients is ongoing. Furthermore, a unifying link between VA and electromechanical coupling may exist, as E_V_ is correlated with different times of the cardiac cycle.

**Materials and Methods:**

This study was a retrospective analysis of a prospectively collected observational database from one tertiary center ICU. We analyzed the relationship between electromechanical dyssynchrony and acute circulatory failure hemodynamics before and after treatment (i.e., fluid expansion, dobutamine, or norepinephrine infusion). The relationship between electromechanical coupling and VA coupling was also investigated. Adult patients with haemodynamic instability were included. Haemodynamic parameters, including arterial pressure, cardiac index, VA coupling, stroke work index/pressure–volume area (SWI/PVA), t-IVT, and Tei's index, were collected before and after treatment. A t-IVT of >12 s/min was classified as intraventricular dyssynchrony.

**Results:**

We included 54 patients; 39 (72.2%) were classified as having intraventricular dyssynchrony at baseline. These patients with baseline dyssynchrony showed a statistically significant amelioration of t-IVT (from 18 ± 4 s to 14 ± 6 s, *p* = 0.001), left ventricular E_V_ [from 1.1 (0.72–1.52) to 1.33 (0.84–1.67) mmHg mL^−1^, *p* = 0.001], VA coupling [from 2 (1.67–2.59) to 1.80 (1.40–2.21), *p* = 0.001], and SWI/PVA [from 0.58 (0.49–0.65) to 0.64 (0.51–0.68), *p* = 0.007]. Patients without baseline dyssynchrony showed no statistically significant results. The improvement in VA coupling was mediated by an amelioration of E_V_. All patients improved their arterial pressure and cardiac index with treatment. The haemodynamic treatment group exhibited no effect on changing t-IVT.

**Conclusion:**

Acute circulatory failure is associated with electromechanical dyssynchrony. Cardiac electromechanical coupling was improved by haemodynamic treatment only if altered at baseline. The improvement of cardiac electromechanical coupling was associated with the improvement of markers of cardiocirculatory efficacy and efficiency (i.e., SWI/PVA and VA coupling). This study was the first to demonstrate a possible link between cardiac electromechanical coupling and VA coupling in patients with acute circulatory failure.

## Introduction

Targeting ventriculo-arterial coupling is a promising approach in patients with cardiocirculatory failure (1–3), because of its intrinsic connection with myocardium energetics and cardiovascular efficiency (4, 5). Ventriculo-arterial coupling is an integrative concept that numerically encompasses the volume/pressure interaction of the left ventricle (LV) with the arterial system, being described as the ratio between the global arterial elastance (E_A_) and left ventricular elastance (E_V_) (3, 6). Several intensive care (ICU) studies have shown the potential of ventriculo-arterial coupling determination for haemodynamic optimisation in different types of circulatory failure (7–9).

Naturally, E_V_ is linked with the LV ejection fraction (LVEF). In a study underlying the limits of LVEF in heart failure, E_V_ was found to be strongly correlated with different times of the cardiac cycle (10)—a relation that was also suggested by other studies (11, 12). This finding opens an interesting research niche regarding the relation between ventriculo-arterial coupling and electromechanical coupling—a subject that has never been studied in critically ill patients. Electromechanical coupling is a parallel study paradigm in haemodynamic research. Several echocardiographic time intervals and parameters are associated with cardiac performance, thus with clinical outcomes (13–16). Tei's index, which is defined as the sum of isovolumic contraction time and relaxation time divided by the ejection time, is described as an index of global myocardial performance and as a prognostic factor in patients presenting with acute myocardial infarction (17). The isovolumic time/heterovolumic time ratio (I/H) index, which is defined as the sum of isovolumic relaxation and contraction time divided by the sum of ejection and filling time, has also been considered a potential indicator of myocardial time yield and efficiency (18). Another marker of heart time efficiency is the total isovolumic time (t-IVT), which is correlated with cardiac activation and performance (19, 20). Prolonged t-IVT is a predictor of future cardiac events after cardiac surgery (21). Reducing the echocardiographic measured time of isovolumic contraction—an indirect marker of electromechanical efficiency—has improved cardiac output and, consequently, heart energetics (22, 23). A correlation between cardiac time intervals and the indicators of systolic function is found in patients admitted to cardiac ICU (24).

Nevertheless, validation of echocardiographic parameters in critically ill patients is in progress (24). Studies evaluating the effect of different haemodynamic ICU treatments (i.e., inotrope, vasopressor, fluid therapy) on cardiac time intervals in patients with acute cardiocirculatory failure are lacking. The clinical role of intraventricular dyssynchrony is unknown in general ICU patients, although it may represent a therapeutic target (22). At present, no study has evaluated the link between electromechanical coupling and ventriculo-arterial coupling in acute circulatory failure, even though answering this question is of clinical and physiological interest. First, identifying the right patients with acute circulatory failure benefitting from haemodynamic interventions is of clinical importance, as the therapeutic arsenal is rather parsimonious, with few and not-so-new available drugs with potential deleterious adverse effects (25). Second, the relationship between ventriculo-arterial and electromechanical coupling may unify two parallel paradigms of haemodynamic research, deepening the understanding of circulatory pathophysiological mechanisms.

The main study objective was to investigate the relationship between electromechanical dyssynchrony and acute circulatory failure and to assess its response to haemodynamic treatment. The secondary objective was to evaluate the relationship between electromechanical coupling and ventriculo-arterial coupling.

## Methods

### Patients and Ethics

This study was a retrospective analysis of a prospectively collected observational database for studying the ventriculo-arterial coupling in the ICU (5). All patients received written information and provided their consent to participate. The study received the approval of the local independent ethics committee, and it was conducted according to the standards of the 1964 Declaration of Helsinki.

This observational study was performed in a cardiovascular ICU at a university hospital. The patients included had haemodynamic instability (MAP < 60 mmHg and/or PAS < 90 mmHg), were aged 18 years or above, and were under controlled positive ventilation. For these patients, the intensivist decided to perform a haemodynamic therapeutic intervention (i.e., fluid expansion, norepinephrine infusion increase, or starting dobutamine infusion) in accordance with the unit's care protocol. The non-inclusion criteria were those who had low echogenicity, a pacemaker, bundle branch block, permanent atrial fibrillation, more than mild aortic regurgitation, or mitral regurgitation, and right heart dysfunction.

### Haemodynamic Parameters

Systolic blood pressure (SAP), diastolic blood pressure (DAP), and mean blood pressure (MAP) were measured using an invasive arterial catheter. Transthoracic echocardiography (CX50 Ultrasound System and an S5-1 Sector Array Transducer, Philips Medical System, Suresnes, France) was performed by a physician blinded to the results of the study. The echocardiographic parameters were calculated as the average of five measurements (regardless of the respiratory cycle). The data were acquired and stored for further analysis. LVEF, end-systolic volume (ESV), and end-diastolic volume (EDV) were calculated using Simpson's method on a four-chamber view. The diameter of the left ventricular outflow tract was measured on a long-axis parasternal view at the time of patient inclusion. The aortic velocity time integral (VTIAo), pre-ejection time and systolic time were measured using a pulsed Doppler on a five-chamber apical view. Stroke volume (SV) (mL) was calculated as VTIAo × aortic area and was expressed as indexed SV (SVi) = SV/body surface area (mL m^−2^). Cardiac index (CI) (L min^−1^ m^−2^) was calculated as SVi × heart rate (HR). The indexed systemic vascular resistance (SVRi) (mmHg mL^−1^ m^−2^) was obtained using the formula SVRi = MAP–central venous pressure/CI. Total arterial compliance (mL mmHg^−1^) was calculated as the ratio between the SV and the arterial pulse pressure.

Ventriculo-arterial coupling is defined as the ratio between E_A_ and left E_V_, with 1 ± 0.36 as the normal value (26, 27). Ventriculo-arterial coupling was calculated using the non-invasive single-beat echocardiographic method, as previously described and recommended by the European Society of Cardiology consensus (3, 12, 28). E_A_ was calculated using the formula E_A_ = 0.9 × SAP/SV, where SAP is systolic arterial pressure, and SV is stroke volume. The indexed stroke work (SW) was calculated as end-systolic pressure (ESP) × SV. The pressure–volume area (PVA), which corresponds to the total energy generated by each cardiac contraction, was calculated using the formula PVA = SW + PE, where PE is the potential energy stored at the end of systole (4). PE was calculated as ESP × ([ESV – V_0_]/2), assuming that V_0_ is negligible compared to the ESV. Using these variables, the SW index/pressure–volume area (SWI/PVA) ratio was further calculated, corresponding to the mechanical efficiency of converting the total mechanical energy (i.e., PVA) available to the left ventricular SW (4).

### Echocardiographic Time Interval Measurement

T-IVT was calculated using the formula t-IVT = 60 – (t-ET + t-FT), where t-ET is the total ejection time, and t-FT is the total filling time (19). A t-IVT higher than 12 s/min was classified as systolic electromechanical inefficiency (i.e., intraventricular dyssynchrony) (24). A 10% improvement in t-IVT after treatment was considered a positive response. The Tei index was calculated as Tei index = isovolumic contraction time + isovolumic relaxation time/ejection time (29). The I/H index was calculated as I/H index = (isovolumic contraction time + isovolumic relaxation time)/(filling time + ejection time) (18).

### Study Procedures

General demographic parameters were collected at the moment of inclusion (i.e., age, gender, type of surgery, and ventilation parameters). Patients were evaluated at two consecutive steps: (1) at the baseline and after therapeutic intervention and (2) after 15 min of haemodynamic stability, which is a change in MAP of <10%. The indication for haemodynamic treatment with fluid expansion, norepinephrine, or dobutamine infusion was left at the discretion of the physician in charge of the patient. All patients were sedated by continuous infusion of propofol and were fully adapted to volume-controlled mechanical ventilation. All patients were mechanically ventilated in a pressure-controlled mode with a positive end-expiratory pressure (PEEP) of 5 cm H_2_O. Ventilator settings (i.e., inspired oxygen fraction, tidal volume, respiratory rate, and PEEP) and propofol infusion rate were not modified during the study period.

### Statistical Analyses

The database was complete; thus, no imputation strategy for missing data was necessary. The normal distribution of variables was assessed using histograms and the Shapiro-Wilk test. Data were expressed as numbers, proportions (in percent), medians [25–75% interquartile range], or as means (± standard deviation), as appropriate. The correlation was assessed using Pearson's or Spearman's tests, as appropriate. Paired data were compared using the Wilcoxon signed rank test.

Logistic regression models were performed to evaluate the relation between the baseline characteristics, the type of hemodynamic intervention, and baseline dyssynchrony status. Logistic regression models were further performed to evaluate the relation between the baseline characteristics, the type of hemodynamic intervention, and the response to the treatment. The responsive patients were defined as those with a decrease of at least 10% of baseline total isovolumic time after treatment. The conditions of validity for the logistic regression were verified, in order to have at least 5–10 events for each independent variable included in the model.

In the absence of previous data, we estimated that a sample size of 40 patients would allow us to demonstrate a correlation between t-IVT and CI or E_A_/E_V_ higher than 0.4 using Spearman's correlation coefficient with a risk alpha of 5% and a power of 80%. Considering a 10% risk of loss or missing data, we decided to include 54 patients.

Statistical analyses were performed using RStudio (version 1.1.447©2009–2018 RStudio, Inc.) and SPSS® (IBM SPSS Statistics for Windows, version 21.0. Armonk, NY, IBM Corp.). A bilateral *p*-value of 0.05 was considered statistically significant.

## Results

Fifty-four patients were included in the study. Their general characteristics are summarized in Table 1. Their mean age was 66 ± 12 years; they were predominantly males (78%), ASA III (75.9%) and IV (20.4%). The most common surgery was valvular surgery (52%), followed by coronary artery bypass graft surgery (19%). Approximately 43% of the patients underwent fluid expansion therapy, and 37 and 20% had norepinephrine and dobutamine infusions, respectively.

**Table 1:** Patients' general characteristics.

**Variable**	**All cohort, *n* = 54**
Age (years), mean ± SD	66 ± 12
Female gender, *n* (%)	12 (22)
SAPS II, mean ± SD	42 ± 14
Type of surgery, *n* (%)	
- Valvular surgery	28 (51.9)
- CABG	10 (18.5)
- Combined surgery	6 (11.1)
- Other cardiac surgery	10 (18.5)
Type of haemodynamic intervention, *n* (%)	
- Fluid expansion	23 (43)
- Norepinephrine infusion	20 (37)
- Dobutamine infusion	11 (20)
Tidal volume (ml/kg), median [IQR]	8 (7, 8)
Deceased, *n* (%)	6 (11.1)
ICU LOS, median [IQR]	3 (2–6)

*SAPS II, simplified acute physiology score II; CABG, coronary artery bypass graft surgery; ICU LOS, intensive care unit length of stay; IQR, 25%−75% interquartile range; SD, standard deviation*.

**Figure 1:**
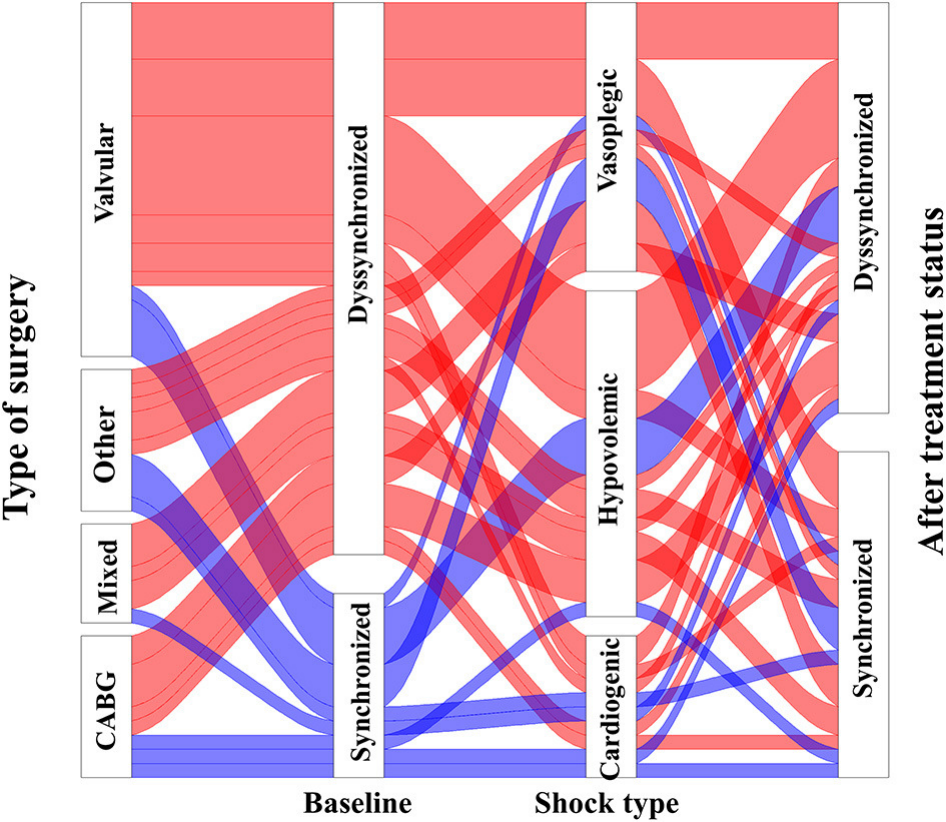
Representation of baseline ventricular dyssynchrony (t-IVT), and its evolution according to baseline and hemodynamic treatment.

The haemodynamic parameters before and after therapeutic intervention for all cohorts are presented in Table 2. 39 (72%) of the 54 patients had a t-IVT over 12 s, and they were classified as having intraventricular dyssynchrony. Based on the logistic regression analysis, no demographic, baseline hemodynamic parameters and type of acute circulatory failure (hypovolemic, vasoplegic, cardiogenic) were associated to dyssynchrony (i.e., a t-IVT over 12 s) (Figure 1, Supplementary Table S1). In the whole cohort, baseline t-IVT was not correlated to CI, LVEF, HR, blood pressure, E_V_, cardiac power, or VA-coupling. The MAP, SVi, CI, and LVEF significantly changed with treatment in all cohorts, without modifying peripheral resistances and HR. The patients with intraventricular dyssynchrony had an indexed EDV of 56 (44–81) ml/m^2^, and the patients without baseline dyssynchrony had an index EDV of 62 (52–96) ml/m^2^, without a statistically significant difference (*p* = 0.161).

**Table 2:** Comparison of haemodynamic parameters before and after interventions.

**Variable**	**Baseline**	**After haemodynamic intervention**	***p-*value**
**HR (bpm), mean** **±SD**			
Overall	82 ± 19	82 ± 19	0.298
No intraventricular dyssynchrony	78 ± 22	81 ± 21	0.234
With intraventricular dyssynchrony	84 ± 18	82 ± 19	0.048
**SAP (mmHg), mean** **±SD**			
Overall	97 ± 18	119 ± 17	<0.0001
No intraventricular dyssynchrony	98 ± 13	118 ± 17	0.001
With intraventricular dyssynchrony	96 ± 20	119 ± 17	<0.0001
**MAP (mmHg), mean** **±SD**			
Overall	67 ± 13	80 ± 12	<0.0001
No intraventricular dyssynchrony	68 ± 10	78 ± 10	0.001
With intraventricular dyssynchrony	67 ± 14	81 ± 13	<0.0001
**DAP (mmHg), mean** **±SD**			
Overall	53 ± 12	61 ± 12	<0.0001
No intraventricular dyssynchrony	52 ± 11	58 ± 11	0.002
With intraventricular dyssynchrony	53 ± 12	61 ± 12	<0.0001
**CI (L min**^**−1**^ **m**^**−2**^**), mean** **±SD**			
Overall	1.76 ± 0.6	2.07 ± 0.6	<0.0001
No intraventricular dyssynchrony	1.67 ± 0.7	1.96 ± 0.7	0.006
With intraventricular dyssynchrony	1.79 ± 0.5	2.11 ± 0.5	<0.0001
**LVEF (%), mean** **±SD**			
Overall	40 ± 14	44 ± 11	<0.0001
No intraventricular dyssynchrony.	37 ± 15	41 ± 12	0.025
With intraventricular dyssynchrony	41 ± 14	45 ± 11	0.002
**SWI/PVA, median [IQR]**			
Overall	0.58 [0.46–0.66]	0.61 [0.5–0.69]	0.005
No intraventricular dyssynchrony	0.58 [0.34–0.67]	0.54 [0.41–0.69]	0.532
With intraventricular dyssynchrony	0.58 [0.49–0.65]	0.64 [0.51–0.68]	0.007
**E**_**A**_ **(mmHg mL**^**−1**^**), median [IQR]**			
Overall	2.14 [1.55–2.83]	2.08 [1.6–2.82]	0.350
No intraventricular dyssynchrony	2.14 [1.42–3.28]	1.95 [1.56–3.48]	0.691
With intraventricular dyssynchrony	2.13 [1.55–2.74]	2.1 [1.61–2.73]	0.395
**E**_**V**_ **(mmHg mL**^**−1**^**), median [IQR]**			
Overall	1.11 [0.76–1.51]	1.37 [0.91–1.69]	<0.0001
No intraventricular dyssynchrony.	1.11 [1.05–1.44]	1.47 [0.97–1.74]	0.112
With intraventricular dyssynchrony	1.1 [0.72–1.52]	1.33 [0.84–1.67]	0.001
**E**_**A**_ **/E**_**V**_ **, median [IQR]**			
Overall	1.9 [1.6–2.5]	1.7 [1.4–2.2]	0.002
No intraventricular dyssynchrony	1.86 [1.16–2.43]	1.67 [1.23–2.35]	0.427
With intraventricular dyssynchrony	2 [1.67–2.59]	1.80 [1.40–2.21]	0.001
**Tei index, mean** **±SD**			
Overall	0.88 ± 0.3	0.73 ± 0.3	0.001
No intraventricular dyssynchrony.	0.56 ± 0.2*	0.66 ± 0.3	0.691
With intraventricular dyssynchrony	1 ± 0.3*	0.76 ± 0.3	<0.0001
**I/H index (s), mean** **±SD**			
Overall	0.36 ± 0.18	0.32 ± 0.2	0.089
No intraventricular dyssynchrony.	0.17 ± 0.08	0.29 ± 0.19	0.006
With intraventricular dyssynchrony	0.44 ± 0.15	0.33 ± 0.22	0.001
**t-IVT (s), mean** **±SD**			
Overall	15.3 ± 6	13.6 ± 6	0.071
No intraventricular dyssynchrony.	8.4 ± 4*	12.7 ± 5	0.006
With intraventricular dyssynchrony	18 ± 4*	14 ± 6	0.001
**t-FT (s), mean** **±SD**			
Overall	27 ± 6	27 ± 6	0.935
No intraventricular dyssynchrony.	33 ± 4*	28 ± 6	0.005
With intraventricular dyssynchrony	25 ± 6*	27 ± 6	0.081
**t-ET (s), mean** **±SD**			
Overall	18 ± 3	19 ± 3	<0.0001
No intraventricular dyssynchrony.	19 ± 5	19 ± 4	0.334
With intraventricular dyssynchrony	17 ± 3	19 ± 3	<0.0001
**Ejection time/filling time ratio, mean** **±SD**			
Overall	0.69 ± 0.2	0.74 ± 0.2	0.088
No intraventricular dyssynchrony.	0.59 ± 0.2*	0.72 ± 0.3	0.041
With intraventricular dyssynchrony	0.73 ± 0.2*	0.75 ± 0.2	0.559

All cohort (overall) and comparatively, patients with intraventricular dyssynchrony vs. patients with no intraventricular dyssynchrony. Comparisons were performed between before (baseline) and after haemodynamic intervention timepoints (shown p-values) and between patients with intraventricular dyssynchrony and patients without intraventricular dyssynchrony at each timepoint (*, Statistically significant difference).

*HR, heart rate; SAP, systolic arterial pressure; MAP, mean systolic pressure; DAP, diastolic arterial pressure; LVEF, left ventricle ejection fraction; t-FT, total filling time; t-ET, total ejection time; t-IVT, total isovolumic time; CI, cardiac index; E_A_, arterial elastance; E_V_, ventricular elastance; Swi, indexed stroke work; PVA, pressure-volume-area*.

### Impact of Haemodynamic Interventions on Total Isovolumic Time

The haemodynamic parameters are presented in Table 2, according to the study group (i.e., with or without baseline intraventricular dyssynchrony) and before and after treatment. Changes of t-IVT was independent of type of hemodynamic treatment (*p* > 0.05, Figure 1, Supplementary Table S2). At baseline, prolonged t-IVT was due to a decreased filling time (33 ± 4 s vs. 26 ± 5 s, *p* < 0.001) and the ejection time being similar between the two groups (18.6 ± 4.6 s vs. 17.6 ± 2.7 s, *p* = 0.505). The baseline ratio of ejection time to filling time was high in patients with intraventricular dyssynchrony (0.73 ± 0.2 vs. 0.59 ± 0.2, *p* = 0.031).

**Figure 2:**
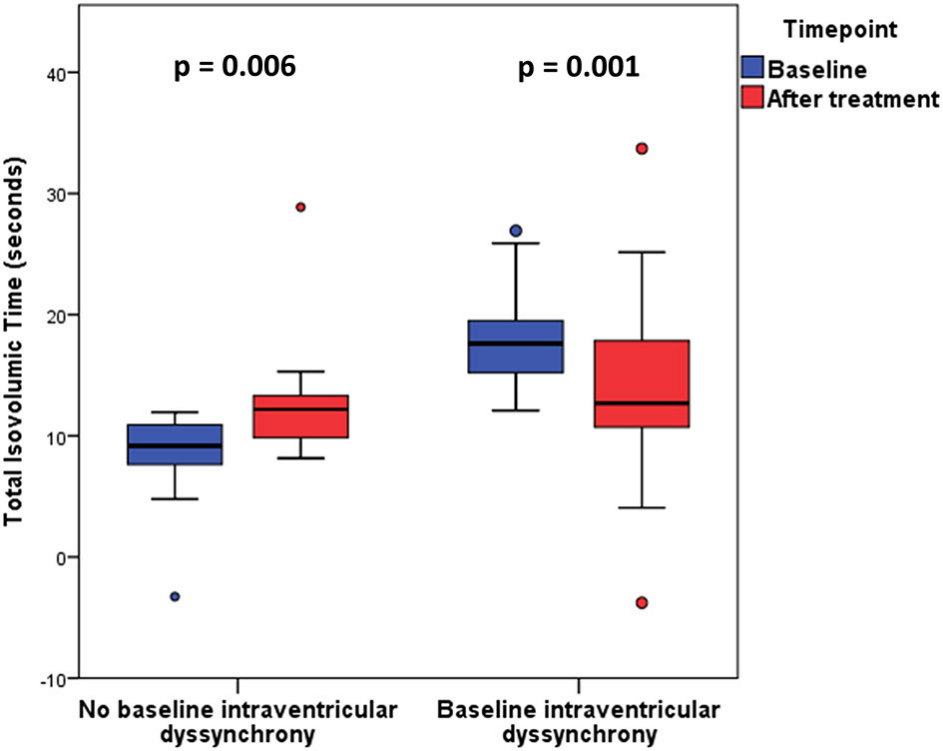
Total Isovolumic time before and after treatment in patients with and without baseline intraventricular dyssynchrony.

**Figure 3:**
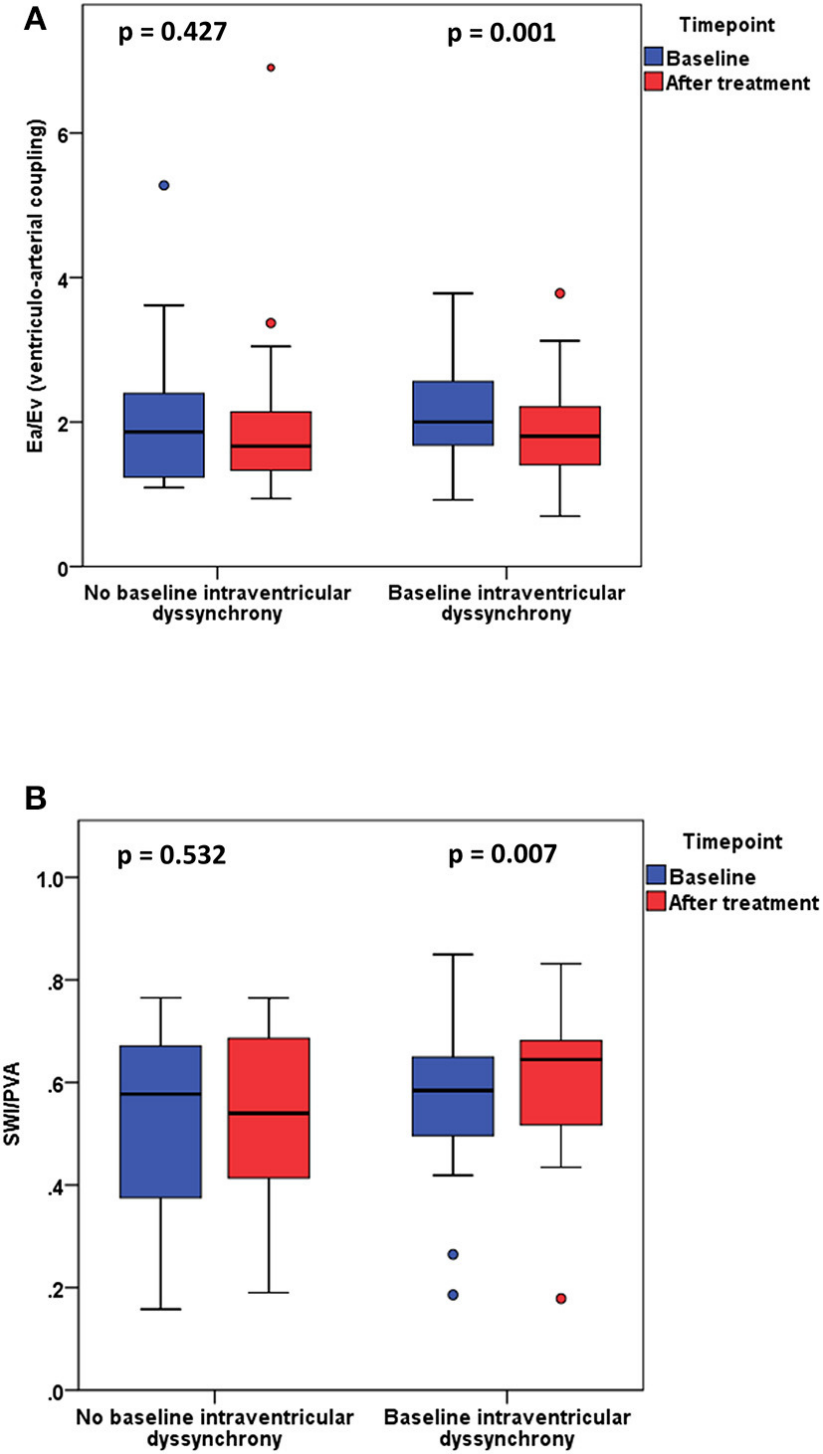
**(A)** Ventriculo-arterial coupling (E_A_/E_V_) before and after treatment in patients with and without baseline intraventricular dyssynchrony. **(B)** Stroke work index/pressure volume area (SWI/PVA) before and after treatment in patients with and without baseline intraventricular dyssynchrony.

Patients with baseline intraventricular dyssynchrony significantly improved their t-IVT (Figure 2, Table 2). This improvement was mainly due to an increase in total ejection time. By contrast, patients without baseline dyssynchrony significantly prolonged their t-IVT with treatment (Figure 2) because of an increase in filling time without changing the ejection time. The type of haemodynamic treatment showed no effect on t-IVT changes (*p* = 0.906). Patients with intraventricular dyssynchrony significantly improved E_V_, ventriculo-arterial coupling (Figure 3A), and SWI/PVA (Figure 3B). The improvement of ventriculo-arterial coupling was due to an increase in E_V_ without changing E_A_.

### Impact of Haemodynamic Interventions on I/H and Tei Indices

The Tei index decreased with haemodynamic treatment (0.83 ± 0.3 vs. 0.77 ± 0.3, *p* = 0.001). The decrease was highly statistically significant in the group with baseline intraventricular dyssynchrony. The Tei-index was correlated with t-IVT at baseline (rho = 0.684, *p* < 0.001) and after treatment (rho = 0.719, *p* < 0.001). The changes in the Tei index were different between the treatment subgroups (*p* = 0.03). The Tei index response seemed higher in the norepinephrine subgroup [−32% (−47; −14)] compared with that in the fluid expansion subgroup [−8% (−30; 14)] and dobutamine subgroup [−3% (−27; 53)].

The I/H index did not change with treatment in the cohort as a whole. Nevertheless, it significantly improved in patients with intraventricular dyssynchrony, whereas it worsened in patients without intraventricular dyssynchrony. I/H index and t-IVT were correlated at baseline (rho = 0.997, *p* < 0.001) and after treatment (rho = 1, *p* < 0.001). The type of haemodynamic treatment was not associated with I/H index changes (*p* = 0.875).

## Discussion

This study demonstrated a link between cardiac electromechanical coupling and ventriculo-arterial coupling in patients suffering from acute circulatory failure. Our results can be summarized as follows: (1) patients suffering from acute circulatory failure exhibited cardiac electromechanical uncoupling, independently of the macro-hemodynamic status, type of shock, and patient's characteristics, (2) the cardiac electromechanical coupling was improved by haemodynamic treatment only if altered at baseline (i.e., in patients with intraventricular dyssynchrony), and (3) the improvement of cardiac electromechanical coupling was associated with improvement of markers of cardiocirculatory efficacy and efficiency (i.e., SWI/PVA and ventriculo-arterial coupling).

Ventriculo-arterial coupling considers the complex relationship between ventricular and arterial system elastances, and it exhibits a strong link with cardiac energetics and cardiac efficiency (3, 4, 30, 31). The mechanism of ventriculo-arterial coupling improvement in our cohort, and specifically for patients with baseline dyssynchrony, was mainly through the amelioration of E_V_. The increase in E_V_ with a steady E_A_ induced the improvement of ventriculo-arterial coupling.

An association between t-IVT and macro-haemodynamic parameters (blood pressure, cardiac output) was observed in patients in the general ICU (24). Baseline t-IVT is able to predict further post-operative events (21). We did not find such an association in our cohort. This discrepancy could be explained by the fact that we studied patients during acute hemodynamic resuscitation phase, where the patients are mostly hemodynamically instable with arterial hypotension and myocardial perfusion mismatch. Several studies have highlighted the complex relationship between ventricular dyssynchrony, aortic blood pressure, coronary flow, and myocardial contractility (32–34). Tavazzi et al. (24) already demonstrated that the underlying disease (i.e., cardiovascular or respiratory) was not associated to ventricular dyssynchrony. As we did not find any association between the type of cardiac surgery and prevalence of ventricular dyssynchrony, we confirmed this point. t-IVT should probably be analyzed as a global marker of ventricular dyssynchrony that reflect whole myocardial performance independently of underlying myocardial disease per se.

In our study, the improvement of t-IVT in patients with baseline dyssynchrony was associated with an improvement in ejection time in contrast with the filling time. At baseline, the ratio between ejection time and filling time was high in the case of dyssynchrony. After haemodynamic intervention, this ratio slightly increased in the dyssynchrony group and significantly increased in the other groups. Given that the t-IVT may be considered as “wasted time” during the heart contraction cycle (18), any improvement of the cardiac electromechanical coupling may be associated with an improvement in cardiac function E_V_. Tavazzi et al. (22) previously demonstrated that patients suffering from cardiogenic shock can be treated with t-IVT-based pacemaker resynchronisation. Although evaluating the effects of each haemodynamic treatment is not an objective of the study, the robustness of t-IVT was suggested by the fact that we observed the same response for the Tei index and I/H index, which are the two known indexes of cardiac efficiency. These indices showed good correlation values.

### Clinical Implications

The study results are of interest, as the Chen's non-invasive determination of ventriculo-arterial coupling does not explore the ventricular loading (3). Our findings confirmed the hypotheses of previous studies that acute circulatory failure can be associated with intraventricular dyssynchrony, and that t-IVT is a potential target for titrating-specific haemodynamic therapy (22). t-IVT has additional value to ventriculo-arterial coupling in patients suffering from acute circulatory failure. We observed a high prevalence of intraventricular dyssynchrony in ICU patients (24), and the cardiologic literature on this subject has already demonstrated an association between electromechanical dyssynchrony and clinical outcomes (11, 13). Studies have suggested an effect on the electromechanical coupling of several haemodynamic treatments.

Several ICU studies considered the titration of haemodynamic treatments based on ventriculo-arterial coupling measurements (7, 9, 35), and algorithms have even been proposed (25, 36). During septic shock, fluid expansion can improve left ventricular relaxation (37). HR modulation improves ventriculo-arterial coupling in patients with septic shock; however, no data are available regarding the echocardiographic time intervals in general ICU patients (38). The interference of norepinephrine infusion with diastolic function has also been noted (39). Tavazzi et al. (23) obtained haemodynamic improvement using echocardiography-guided pacemaker optimisation of t-IVT—a method that is not easily available at bedside. In our study, the changes in t-IVT were independent of the haemodynamic treatment used for acute circulatory failure, suggesting a role for the relationship between myocardial perfusion, myocardial contractility, and ventricular dyssynchrony in acute circulatory failure.

A future perspective is the evaluation of targeting t-IVT in patients with septic shock. The optimisation of ventriculo-arterial coupling is feasible and associated with haemodynamic and tissue perfusion improvement (40). Considering septic cardiomyopathy (41), t-IVT determination may provide further understanding of the complex pathophysiology and management aspects of critically ill patients. Furthermore, our results suggested that haemodynamically unstable patients with prolonged t-IVT benefit more from further haemodynamic interventions. In the era of “less is more” ICU approach, this finding is of clinical significance in acute settings. As it suggests, t-IVT may be used to discern patients in whom rapid and further therapeutic interventions must be administered, from patients for whom a temporary conservatory approach must be selected.

### Limitations

Our study can be discussed on several points. This study is an observational study that demonstrated association but not causal relation. One limitation is the heterogeneity and the limited number of patients in the study. We performed a sensitivity analysis to determine which factors were associated with ventricular dyssynchrony. None of baseline demographic and hemodynamic parameters were associated to ventricular dyssynchrony. Moreover, we were unable to demonstrate association between the type of hemodynamic treatment and ventricular dyssynchrony. The changes in t-IVT were independent of the type of haemodynamic treatment, and the proportion of each haemodynamic intervention did not differ between the two groups of patients. The aforementioned relationship between blood pressure, coronary blood perfusion, and ventricular dyssynchrony may explain (in part) our results, and the fact that during acute phase of shock the main determinant of ventricular dyssynchrony may be heart blood perfusion and cardiovascular interactions. The sample size of the present study may be limited but we performed a sample size calculation based on the study of Tavazzi et al. (34). Previous studies evaluating the t-IVT and/or the hemodynamic effect had sample size close to this of the present study (7, 21). In addition, the purpose of our work was not to precisely describe the individual effects of each haemodynamic treatment on the components of ventriculo-arterial coupling or t-IVT. Several studies already considered and described these effects (7–9). Another point of discussion is the risk of mathematical coupling between t-IVT and EV because we used Chen's method, which includes systolic ejection time. However, t-IVT also includes isovolumic relaxation time, which is not only fully determined by ejection time. Moreover, the t-IVT formula includes the filling time. We observed the changes of each component (i.e., systolic and filling time) of this index. In addition, we also evaluated other indices, that is, the Tei and I/H indexes, to broaden our perspective. The present study may be considered as the first one to highlight an association between ventricular dyssynchrony and ventriculo-arterial coupling in acute circulatory failure. Further studies may confirm our results, and they will focus on the mechanisms involved in ventricular dyssynchrony during acute circulatory failure.

## Conclusion

Acute circulatory failure is associated with electromechanical dyssynchrony (i.e., prolonged t-IVT). Patients with electromechanical dyssynchrony are those who further benefit from haemodynamic treatment in terms of cardiac efficacy and efficiency. T-IVT may represent a potential target for haemodynamic monitoring and optimisation in patients suffering from acute circulatory failure.

## Data Availability Statement

The raw data supporting the conclusions of this article will be made available by the authors, without undue reservation.

## Ethics Statement

The studies involving human participants were reviewed and approved by the Comité de Protection des Personnes Nord-Ouest II CHU - Place V. Pauchet, 80054 AMIENS Cedex 1. The patients/participants provided their written informed consent to participate in this study.

## Author Contributions

SA and P-GG conceived the study, analyzed the data, and drafted the manuscript. VC and P-GG collected the data. BP, MN, and BB provided a critical revision of the manuscript. All authors contributed to the article and approved the submitted version.

## Conflict of Interest

The authors declare that the research was conducted in the absence of any commercial or financial relationships that could be construed as a potential conflict of interest.

## Publisher's Note

All claims expressed in this article are solely those of the authors and do not necessarily represent those of their affiliated organizations, or those of the publisher, the editors and the reviewers. Any product that may be evaluated in this article, or claim that may be made by its manufacturer, is not guaranteed or endorsed by the publisher.
