# Supplementary material for: Role of Electromechanical Dyssynchrony Assessment During Acute Circulatory Failure and Its Relation to Ventriculo-Arterial Coupling
Source: Front Cardiovasc Med. 2022 Jun 21;9:907891. doi: 10.3389/fcvm.2022.907891 (PMC9253504; doi:10.3389/fcvm.2022.907891)
Supplement: Supplementary file 1 [file Table_1.docx]

Supplementary table 1. Univariate logistic regression analyses with baseline dyssynchrony status as dependent variable considering the baseline patients’ characteristics

| **Variables** | ***OR (CI_95%_ )*** | ***p-value*** |
| --- | --- | --- |
| *Comorbidities* |  |  |
| - High blood pressure - Chronic heart failure - Valvular disease - COPD - Smoking - Diabetes - Dyslipidaemia - Chronic kidney disease | 3 (0.8-11)  0.9 (0.2-3)  1.9 (0.6-6.5)  0.5 (0.08-3.6)  0.3 (0.1-1.1)  1 (0.3-4.1)  0.5 (0.1-1.6)  2.5 (0.3-23) | 0.097  0.856  0.283  0.527  0.080  0.910  0.215  0.407 |
| *Type of Surgery* |  |  |
| - Valvular - CABG - Mixed - Other | 1.2 (0.4-4)  0.9 (0.2-3.9)  2.5 (0.3-23)  0.5 (0.1-2.1) | 0.761  0.862  0.407  0.345 |
| *Type of acute circulatory failure* |  |  |
| - Hypovolemic - Vasoplegic - Cardiogenic | 1.5 (0.4-5.4)  1.2 (0.4-4.4)  0.4 (0.1-1.4) | 0.494  0.727  0.151 |
| Abbreviations: OR – odd ratio; COPD – chronic pulmonary obstructive disease; CABG – coronary artery bypass graft. | | |

Supplementary table 2. Univariate logistic regression analyses with after-treatment dyssynchrony response* as dependent variable, considering the baseline patients’ characteristics

| **Variables** | ***OR (CI_95%_ )*** | ***p-value*** |
| --- | --- | --- |
| *Comorbidities* |  |  |
| - High blood pressure - Chronic heart failure - Valvular disease - COPD - Smoking - Diabetes - Dyslipidaemia - Chronic kidney disease | 0.8 (0.2-2.8)  0.8 (0.3-2.7)  1.3 (0.5-3.9)  0.2 (0.02-2.1)  0.7 (0.2-2.2)  2.6 (0.7-9)  1.6 (0.5-4.6)  1.4 (0.3-6.9) | 0.750  0.770  0.586  0.191  0.580  0.135  0.414  0.686 |
| *Type of Surgery* |  |  |
| - Valvular - CABG - Mixed - Other | 0.9 (0.3-2.5)  1 (0.3-4)  2.8 (0.5-16)  0.6 (0.2-2.5) | 0.786  1  0.239  0.486 |
| *Type of acute circulatory failure* |  |  |
| - Hypovolemic - Vasoplegic - Cardiogenic | 1.9 (0.6-5.6)  0.7 (0.2-2.2)  0.8 (0.2-3) | 0.270  0.574  0.736 |
| Abbreviations: OR – odd ratio; COPD – chronic pulmonary obstructive disease; CABG – coronary artery bypass graft. | | |

*The responsive patients were defined as those with a decrease of at least 10% of baseline total isovolumic time after treatment
